# Phosphoproteome analysis during larval development and metamorphosis in the spionid polychaete *Pseudopolydora vexillosa*

**DOI:** 10.1186/1471-213X-11-31

**Published:** 2011-05-25

**Authors:** Kondethimmanahalli H Chandramouli, Flora SY Mok, Hao Wang, Pei-Yuan Qian

**Affiliations:** 1 KAUST Global Collaborative Research Program, Division of Life Science, Hong Kong University of Science and Technology, Hong Kong SAR, China

## Abstract

**Background:**

The metamorphosis of the spionid polychaete *Pseudopolydora vexillosa *includes spontaneous settlement onto soft-bottom habitats and morphogenesis that can be completed in a very short time. A previous study on the total changes to the proteome during the various developmental stages of *P. vexillosa *suggested that little or no *de novo *protein synthesis occurs during metamorphosis. In this study, we used multicolor fluorescence detection of proteins in 2-D gels for differential analysis of proteins and phosphoproteins to reveal the dynamics of post-translational modification proteins in this species. A combination of affinity chromatography, 2D-PAGE, and mass spectrometry was used to identify the phosphoproteins in pre-competent larvae, competent larvae, and newly metamorphosed juveniles.

**Results:**

We reproducibly detected 210, 492, and 172 phosphoproteins in pre-competent larvae, competent larvae, and newly metamorphosed juveniles, respectively. The highest percentage of phosphorylation was observed during the competent larval stage. About 64 stage-specific phosphoprotein spots were detected in the competent stage, and 32 phosphoproteins were found to be significantly differentially expressed in the three stages. We identified 38 phosphoproteins, 10 of which were differentially expressed during metamorphosis. These phosphoproteins belonged to six categories of biological processes: (1) development, (2) cell differentiation and integrity, (3) transcription and translation, (4) metabolism, (5) protein-protein interaction and proteolysis, and (6) receptors and enzymes.

**Conclusion:**

This is the first study to report changes in phosphoprotein expression patterns during the metamorphosis of the marine polychaete *P. vexillosa*. The higher degree of phosphorylation during the process of attaining competence to settle and metamorphose may be due to fast morphological transitions regulated by various mechanisms. Our data are consistent with previous studies showing a high percentage of phosphorylation during competency in the barnacle *Balanus amphitrite *and the bryozoan *Bugula neritina*. The identified phosphoproteins may play an important role during metamorphosis, and further studies on the location and functions of important proteins during metamorphosis are warranted.

## 1.0. Background

*Pseudopolydora vexillosa *is a subtropical spionid polychaete that releases young larvae into the water column, where they develop to competency before settlement. Larval growth involves the formation of segment-specific structures, such as chaetae, and the addition of terminal chaetigers. Metamorphosis of the larvae is a gradual process that does not require substantial development of juvenile organs [1]. When competent larvae find a suitable habitat, they discard their swimming chaetae and secrete mucus while burrowing into the sediment. During metamorphosis into benthic juveniles, the larvae build tubes around themselves while redirecting their palps toward the anterior portion [2]. *P. vexillosa *can complete settlement and metamorphosis within three hours after attaining competency. Larval development of spioniform polychaetes is well studied [2,3]; even so, there have been few detailed studies on the molecular mechanisms of larval metamorphosis, particularly on the dynamics of protein modification. Proteomic analysis is regarded as a powerful approach to the large-scale investigation of proteins [4,5]. Our previous studies using 2-D gel electrophoresis (2-DE) have demonstrated that little or no *de novo *protein synthesis occurs during the metamorphosis of *P. vexillosa *[6]. In this study, we conducted a large-scale analysis of the post-translation modification (PTM) of the proteins that may play a key role in metamorphosis. In contrast to in insects and amphibians, metamorphosis in many marine polychaetes is a quick process and can take place in less than 24 hr [7,8]. To gain good understanding of the most important cellular processes at the molecular level, an understanding of protein modification is required [9]. However, the global analysis of phosphoproteins is rather challenging, because only a small fraction of a particular protein may be phosphorylated at a given time and often in an insufficient quantity for MS analysis. In addition, many regulatory or signaling proteins are present in low abundance in cells, which further complicates their identification [10]. A common approach to overcoming these problems is affinity purification and protein enrichment, which involves affinity enrichment of intact phosphorylated proteins, which are subsequently separated by 2-DE, and the detection by specific phosphoprotein staining and identification by mass spectrometry. A combination of affinity chromatography, 2-DE, and mass spectrometry to identify phosphoproteins reduces the complexity and increases the sensitivity of the analysis. As the purpose of this study was to identify abundantly and differentially expressed phosphorylated proteins in *P. vexillosa *during metamorphosis, sequential fluorescence detection of proteins and phosphoproteins in 2-DE gels for differential analysis of proteins in three developmental stages was used.

## 2.0. Results

### 2.1. Sample complexity and potential problems in protein separation by 2-DE

Our previous analysis of the proteome during larval metamorphosis in *P.vexillosa *was carried out using a broad-range IPG strip (pI 3-10) [6]. We found aggregation of proteins spots on 2-DE gels because of the poor resolution of the proteins. This was mainly because of sample complexity and polysaccharide contamination. In the current study, we improved the protein separation method and reduced the sample complexity by modifying the sample preparation and separation methods. First, larval protein samples were purified by a 2-D clean up kit (BioRad, USA), which not only desalted the sample but efficiently removed polysaccharides and other contaminants from the sample. Secondly, the phosphoproteins were enriched by affinity purification and the elution was stained with a phosphoproteins-specific fluorescent dye, ProQ Diamond (Invitrogen, USA), to check the purity of the phosphoproteins. Finally, the purified phosphoproteins were used for 2-DE using a narrow-range IPG strip (pI 4-7), which provides better separation than a broad range IPG strip (pI 3-10) and reduces sample complexity.

Specific spots were selected for PDQuest analysis and mass spectrometry based on their reproducibility among three replicate gels. Some spots showed differential expression in one replicate but failed to reproduce in other replicates. These spots were not subjected to analysis. In addition, phosphoproteins spots of interest should have sufficient quantity for MS analysis.

### 2.2. Proteome and phosphoproteome profile of *P. vexillosa*

**Figure 1:**
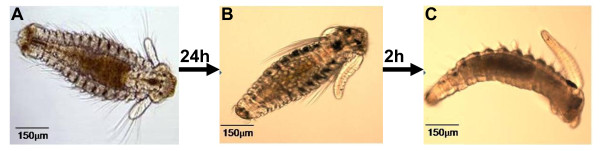
**Developmental stages of the spionid *Pseudopolydora vexillosa***. Three developmental stages were chosen for proteomic analysis: (A) pre-competent larvae, (B) competent larvae, and (C) newly metamorphosed juveniles.

**Figure 2:**
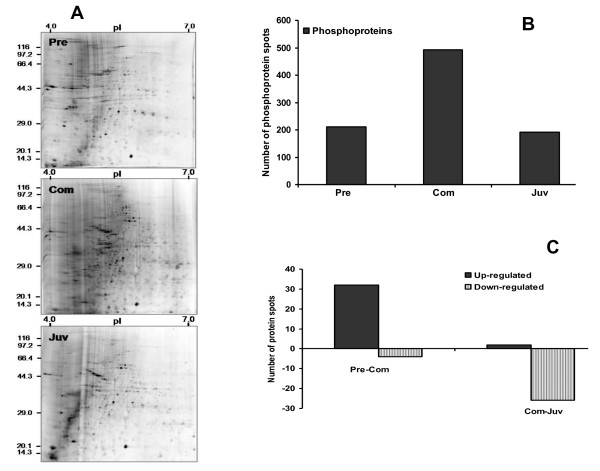
**Representative phosphoproteome gels of *P. vexillosa***. Pre: pre-competent larvae, Com: competent larvae, Juv: newly metamorphosed juveniles. (A) The protein extracts were separated on narrow-range IPG strips (pH/p*I *4-7) followed by 12.5% 2-DE and stained with the phosphoprotein-specific stain ProQ Diamond. (B) Number of phosphoprotein spots reproducibly detected in the three developmental stages. (C) Number of up-regulated and down-regulated phosphoprotein spots (Student's *t*-test (*p *< 0.01, n = 3).

**Figure 3:**
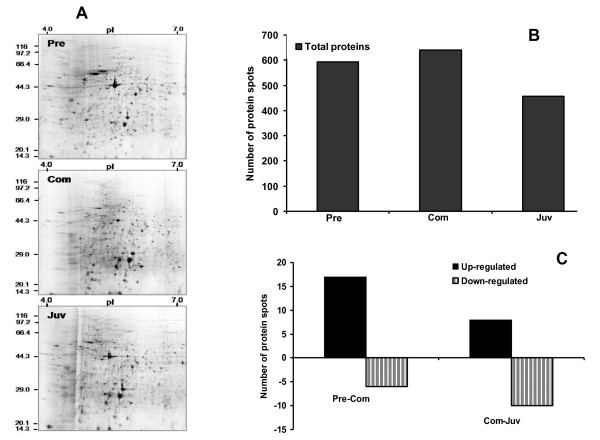
**Representative total proteome gels of *P. vexillosa***. Pre: pre-competent larvae, Com: competent larvae, Juv: newly metamorphosed juveniles. (A) After 2-DE, the gels were stained with the total protein stain Sypro Ruby. (B) Number of protein spots reproducibly detected in the three developmental stages. (C) Number of up-regulated and down-regulated total protein spots. Differentially expressed spots showed a greater than two-fold change and a significant difference between stages (Student's *t*-test (*p *< 0.01, n = 3).

**Figure 4:**
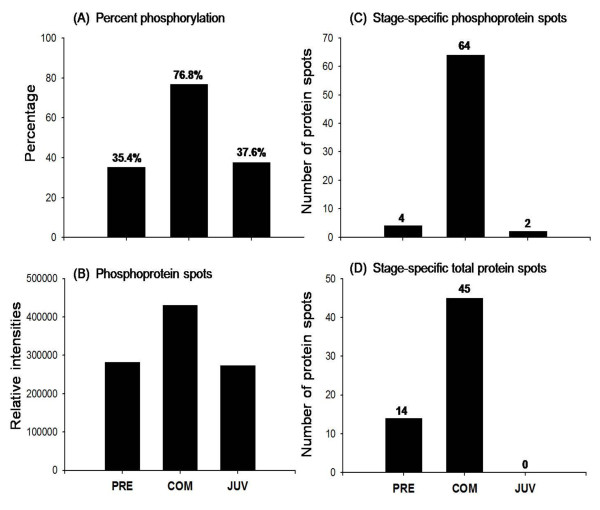
**Relative intensity and stage-specific phosphoproteins in successive developmental stages of *P. vexillosa***. (A) Percentage of phosphorylated proteins, (B) overall relative intensity of the phosphoproteome, (C) number of stage-specific phosphoprotein spots, and (D) number of stage-specific total protein spots (Student's *t*-test (*p *< 0.01, n = 3).

The phosphoproteome 2-D gels of the three developmental stages (Figure 1) were analyzed with PDQuest software. Representative 2-DE gels of larval developmental stages sequentially stained with Pro-Q Diamond and Sypro Ruby fluorescent dyes are shown in Figures 2A and 3A respectively. In the PRECOM, COM, and JUV stages, 210, 492, and 172 phosphoprotein spots (Figure 2B) and 594, 640, and 457 protein spots (Figure 3B) were respectively detected. We observed twice as many phosphorylated spots (76% of all protein spots) in the COM stage than in the PRECOM (35%) or JUV (37%) stages (Figure 2B). The COM stage also had the highest overall relative phosphoproteome intensity, which was at least 1.5-fold higher than that of the PRECOM or JUV stages (Figures 4A and 4B). In the COM stage, we detected 64 stage-specific phosphoprotein spots and 45 stage-specific total protein spots, but only four stage-specific phosphoprotein and 14 total protein spots were detected in the PRECOM stage and only two stage-specific phosphoprotein spots were detected in the JUV stage (Figures 4C and 4D).

### 2.3. Differentially expressed phosphoproteome dynamics

**Figure 5:**
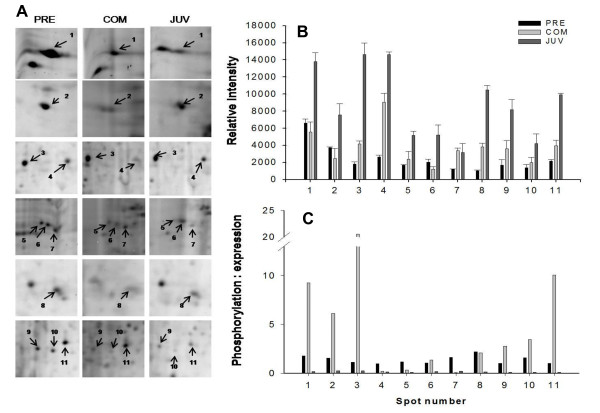
**Close view of phosphoprotein spots (spots 1-11) differentially expressed (indicated by arrows) during metamorphosis in *P. vexillosa***. (A): Enlarged phosphoprotein gels (Pre: pre-competent larvae, Com: competent larvae, Juv: juveniles). (B) Relative intensity of the differentially regulated phosphoproteins. (C) Phosphoprotein-to protein intensity ratios.

**Figure 6:**
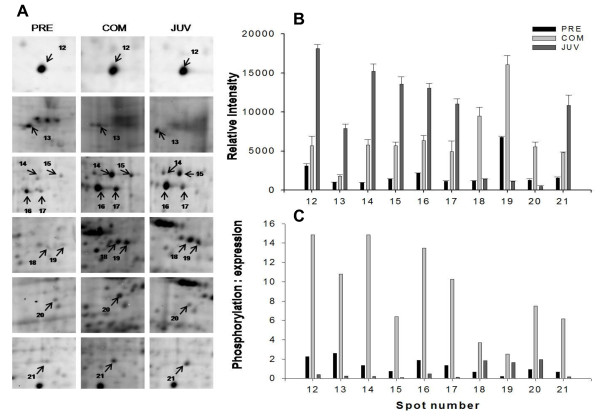
**Close view of phosphoprotein spots (spots 12-21) differentially expressed (indicated by arrows) during metamorphosis in *P. vexillosa***. (A) Enlarged phosphoprotein gels (Pre: pre-competent larvae, Com: competent larvae, Juv: juveniles). (B) Relative intensity of the differentially regulated phosphoproteins. (C) Phosphoprotein-to-protein intensity ratios.

**Figure 7:**
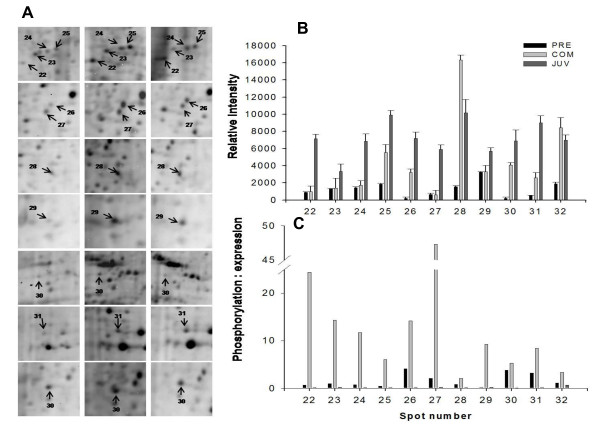
**Close view of phosphoprotein spots (spots 22-32) differentially expressed (indicated by arrows) during metamorphosis in *P. vexillosa***. (A) Enlarged phosphoprotein gels (Pre: pre-competent larvae, Com: competent larvae, Juv: juveniles). (B) Relative intensity of the differentially regulated phosphoproteins. (C) Phosphoprotein-to-protein intensity ratios.

During the transition between the PRECOM and COM stages, 32 phosphoprotein spots were up-regulated and four spots were down-regulated. In contrast, two were up-regulated and 26 down-regulated during the transition between COM and JUV (see the right panel of Figure 2C). A similar trend of differential expression was observed when the gels were stained with Sypro Ruby dye to identify the total proteins: 17 total proteins spots were up-regulated and six spots were down-regulated in the transition from the PRECOM to the COM larvae, whereas eight spots were up-regulated and 10 were down-regulated during the transition between COM and JUV (see the right panel of Figure 3C). About 32 phosphoprotein spots that were expressed in all three stages exhibited significant differences in spot intensity, with 28 spots having a higher phosphorylation intensity in the COM stage, as shown in Figures 5A, 6A, and 7A. To determine whether these spots were truly differentially phosphorylated or whether their phosphoprotein spot intensity merely changed due to differences in the total protein expression, we compared the intensity of the total protein spots with that of the phosphoprotein spots (phosphoprotein: total protein intensity ratio). The results showed that 30 out of the 32 spots (Figures. 5B, C, 6B, C, 7B, and 7C) displayed increased phosphorylation intensity in the COM stage compared with in the PRECOM and JUV stages, suggesting that a high degree of phosphorylation occurred when the larvae attained competence and that dephosphorylation subsequently occurred during metamorphosis.

### 2.4. Affinity purification and identification of abundant phosphoproteins and differentially expressed proteins

**Figure 8:**
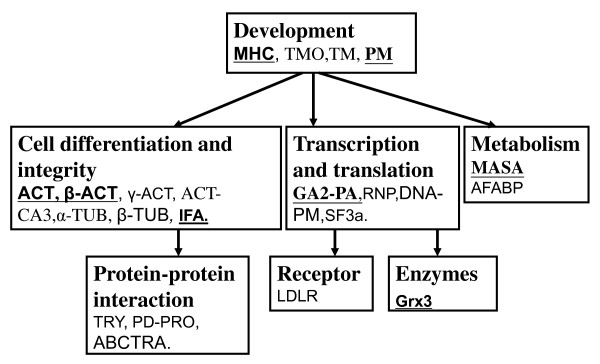
**Functional classification of the identified phosphoproteins**. The differentially expressed proteins are shown in bold and underlined.

GenBank and the EST database currently do not include the genomic sequence of *P. vexillosa*. By coupling a 454 pyrosequencing platform and conducting gene assemblage using the GS *de novo *assembler program, a transcriptome database containing 13,554 contiguous sequences (contigs and isotigs) and 71,941 singlet sequences were generated. The pyrosequencing data were submitted to Sequence Reads Archive (SRA) of NCBI (National Center of Biotechnology Information) with the accession number SRA030597.1. The protein PMF and MS/MS data were then searched against this database. In total, we identified 38 phosphoproteins, 28 of which occurred in abundant phosphorylated spots and 10 in differentially phosphorylated spots, as shown in Tables 1 and 2. In these 38 identified phosphoproteins, 21 phosphoproteins were identified by the transcriptome database of *P. vexillosa*, of which 13 were abundant phosphorylated proteins and eight were differentially regulated phosphorylated spots. An additional 17 phosphoproteins were identified using the NCBInr database. Of these, 15 were abundant phosphorylated spots and two were differentially regulated protein spots. The observed MW and pI values of the identified proteins were very close to the theoretical values derived from a search of the MASCOT database, but the MWs and pIs of a few proteins, including those in spots 1, 28, 29, 31, 40, 41, 45, and 50 in the gel deviated from these values, presumably due to post-translational modification. Many of the proteins identified in the *P. vexillosa *transcriptome database were also identified in the NCBInr database with high confidence scores, reflecting the accuracy of the protein identification. Interestingly, several differential spots were identified as the same protein or isoforms of the same cytoskeleton proteins, such as alpha-tubulins (α-TUB) (spots 46, 47, 48, 50, and 56); beta-tubulins (β-TUB) (spot 56); actins (ACT) (spots 15 and 43); beta-actins (β-ACT) (spots 32, 34, 42, and 55); actin, cytoplasmic A3 (ACT-CA3) (spots 57 and 59); gamma-actin (γ-ACT) (spot 58); intermediate filamant A (IFA) (spots 29 and 44); myosin heavy chain (MHC) (spots 27, 28, and 37); paramyosin (PM) (spot 5); and tropomyosin (TM) (spot 60). Tubulins and actins accounted for 36% of the spots identified, possibly due to the prevalence of different isoforms caused by post-translational modification, in particular phosphorylation or protein degradation during larval metamorphosis. We also identified phosphoproteins related to transcription and translation processes, including GA20008-PA (GA2-PA) (spot 6), ribonucleoprotein (RNP) (spot 33), DNA primase (DNA-PM) (spot 41), and splicing factor 3A (SF3a) (spot 49). In addition, enolase-phosphatase E1 (MASA) (spot 1), polydom protein (PD-PRO) (spot 41), ABC transporters (ABCTRA) (spot 41), epsilon trypsin (TRY) (spot 52), glutaredoxin 3 (Grx3) (spot 3), a low-density lipoprotein receptor (LDLR) (spot 54), and fatty acid-binding protein 4, adipocyte (AFABP) (spot 11) were identified as phosphoproteins regulated during metamorphosis. Among the 10 differentially expressed phosphoproteins identified, MASA, Grx3, GA2-PA, AFABP, ACT, MHC, and IFA showed a higher degree of phosphorylation in the COM stage and significantly decreased expression in the JUV stage. However, the expression of PM followed the opposite trend, as its phosphorylation decreased in the COM stage but increased during metamorphosis. We grouped the phosphoproteins into six biological process categories (see Figure 8): (1) development, (2) cell differentiation and integrity, (3) transcription and translation, (4) metabolism, (5) protein-protein interactions and proteolysis, and (6) receptors and enzymes.

**Table 1:** Identification of Differentially Expressed Proteins in *P. vexillosa *during Metamorphosis by MALDI-TOF MS

**Spot No**.	**Acc No.**^**a)**^	**Protein name **^**b)**^	**MW(kDa)**^**c) **^**Obs./Theo**.	**pI**^**c) **^**Obs./Theo**.	PM/SC (%)	Phosphorylation variation ratios			Biological process
							
						PRE	COM	META	
1	Contig08739_26/gi|224049395	Enolase-phosphatase E1	230/13	3.7/4.2	13/47	1.78	9.25	0.19	Amino-acid biosynthesis

3	GG70GXE04J1FKM_1/gi|198433617	Glutaredoxin	29/25	4.5/9.6	10/19	1.12	20.53	0.27	Redox metabolism, oxidative stress

5	Contig06643_7/gi|126116628	Paramyosin	34/30	5.5/5.1	10/38	1.17	0.33	0.09	Myofibril assembly, muscle contraction

6	gi|91085405	GA20008-PA	32/25	5.8/10	6/25	1.07	1.37	0.18	RNA processing

11	GG7OGXE04JZMIN_9/gi|194214808	Fatty acid binding protein 1, adipocyte	30/20	6.0/8.2	10/44	1.01	10.05	0.11	Energy storage, inflammation

15	GG70GXE04JCRO8_1/gi|37528876	actin	14/10	5.7/4.9	6/44	0.74	6.38	0.08	Cellular structure

27	Contig00736_21/gi|189007782	Myosin heavy chain	66/100	5.6/5.1	18/15	2.09	47.30	0.20	Myogenesis, muscle contraction

28	Contig00736_21/gi|189007782	Myosin heavy chain	63/109	5.1/5.8	8/14	0.82	2.05	0.16	Myogenesis, muscle contraction

29	Contig02680_11/gi|4468655	Intermediate Filament A	60/57	5.5/5.3	9/26	0.12	9.27	0.25	Structural integrity, cell motility

32	gi|182623856	Beta actin	18/25	5.6/5.3	7/34	1.07	3.32	0.69	Cellular structure

a) Accession numbers are from the NCBInr and in-house *P. vexillosa *transcriptomic databases. b) For positive identification, the score had to be over the significance threshold (p < 0.05). c) Observed (Obs.) MW and p*I *values were estimated from 2-DE gels and Theoretical (Theo.) MW and p*I *values were derived from a database search by MASCOT. PM: number of peptides matching the protein sequence; SC: sequence coverage.

**Table 2:** Identification of abundant phosphoproteins in *P. vexillosa *during metamorphosis by MALDI-TOF MS.

**Spot No**.	**Acc No.**^**a)**^	**Protein name **^**b)**^	**MW(kDa)**^**c) **^**Obs./Theo**.	**pI**^**c) **^**Obs./Theo**.	PM/SC (%)	Biological process
33	Contig0667618/gi|291243919	Nuclear ribonucleoprotein	36/34	6.5/6.5	16/22	rRNA processing

34	Contig56810076	Beta-actin	20/19	5.7/5.3	10/28	Cellular structure protein

35	gi|149912747	ABC transporter	33/33	5.8/5.4	7/29	Hormone response regulation

36	gi|116740271	Alpha-tubulin	43/43	6.9/5.9	10/18	Cytokinesis, cell division

37	GG70GXE04JMNIB_2/gi|212449	Myosin heavy chain	60/15	6.6/5.0	03/44	Myogenesis, muscle contraction

38	gi|131573157	Alpha-tubulin	35/45	6.5/5.7	7/25	Cytokinesis, cell division

39	gi|161072	Alpha-tubulin	40/50	5.7/4.9	9/10	Cytokinesis, cell division

40	GG70GXE04H5QQL_10/gi|116250948	Conserved hypothetical protein	40/16	5.5/5.6	4/25	Structural reorganization

41	Contig04504_14/gi|4481960	Polydom protein	40/15	5.5/4.7	12/21	Protein-protein interaction, adhesion

42	F5K2Q4C01CF4OT_4/gi|467215	Actin beta	50/41	5.4/5.5	11/33	Cellular structure protein

43	GG70GXE04JCRO8_11/gi|37528876	Actin	50/41	4.3/5.5	11/43	Cellular structure protein

44	Contig02678_14/gi|4468655	Intermediate Filamant A	70/71	5.6/5.5	16/20	Structural integrity, cell motility

45	gi|169824521	DNA primase	50/69	6.0/6.2	9/17	DNA replication

46	gi|161072	Alpha-tubulin	48/50	6.0/6.4	5/49	Cytokinesis, cell division

47	gi|38047815	Alpha Tub84B	18/28	5.4/5.3	14/25	Cytokinesis, cell division

48	gi|38047815	Alpha Tub84B	18/28	5.4/5.3	9/25	Cytokinesis, cell division

49	Contig10935_1/gi|168761509	Splicing factor 3A subunit 1	16/10	5.6/10	4/46	mRNA processing, splicing

50	gi|131573157	Alpha tubulin	15/10	5.7/5.7	8/57	Cytokinesis, cell division

51	gi|197129678	Tubulin beta 2	6/26	4.5/4.4	4/21	Cytokinesis, cell division

52	gi|999627	Epsilon trypsin	17/9	4.8/6.6	5/46	Proteolysis

53	Contig06726_6/gi|148225538	Tyrosine 3-monooxygenase	35/30	4.5/4.7	4/26	Muscle contraction

54	Contig13459_11/gi|4481960	Low-density lipoprotein receptor	33/22	4.5/4.6	11/31	**C**holesterol homeostasis

55	gi|78190577	Beta-tubulin	55/43	5.3/5.7	11/37	Cytokinesis, cell division

56	gi|159400261	Alpha-tubulin	60/47	5.3/5.2	5/18	Cytokinesis, cell division

57	Contig56810076/gi|37528876	Actin, cytoplasmic	53/45	5.5/5.3	9/28	Cellular structure protein

58	gi|178045	Gamma-actin	29/26	5.5/5.7	12/25	Cellular structure protein

59	Contig56810077/gi|37528876	Actin, cytoplasmic A3	32/45	5.7/5.5	7/18	Cellular structure protein

60	gi|47117349	Tropomyosin	35/39	4.5/4.6	8/19	Muscle degeneration and differentiation

a) Accession numbers are from the NCBInr and in-house *P. vexillosa *transcriptomic databases. b) For positive identification, the score had to be over the significance threshold level (p < 0.05). c) Observed (Obs.) MW and p*I *values were estimated from 2-DE gels and Theoretical (Theo.) MW and p*I *values were derived from a database search by MASCOT. PM: number of peptides matching the protein sequence; SC: sequence coverage.

### 2.5. Confirmation of cytoskeleton protein abundance by 2-DE Western Blot

**Figure 9:**
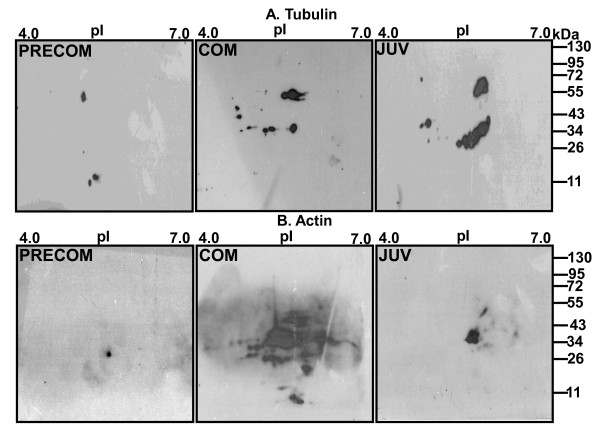
**2-DE Western blot analysis during metamorphosis in *P. vexillosa***. Two-dimensional gel and immunoblot of tubulin **(A) **and actin **(B) **of *P.vexillosa *(Precom: pre-competent larvae, Com: competent larvae, Juv: juveniles) probed with anti-tubulin and anti-actin monoclonal antibodies and developed by ECL western blotting analysis system.

Several spots identified by mass spectrometry were isoforms of cytoskeleton proteins such as tubulin and actin. 2-DE western blots revealed that many of the isoforms of tubulin (upper panel of Figure 9) and actin (lower panel of Figure 9) had molecular weights and pI values (tubulin: Mr ~75-35/pI 5.2-6.4 and actin: Mr ~60-20/pI 5.0-6.0) close to those reported in Tables 1 and 2. Both the α-tubulin (Mr 70-55/pI 4.5-6.0) and β-tubulin (Mr 45-25/pI 4.5-6.0) isoforms were captured in all three developmental stages. Spots were observed in COM and JUV stages of several additional isoforms (pI; 4.5-4.7). Only a few visible tubulin isoforms were found in PRECOM stage, whereas the abundance significantly increased in the COM and JUV stages. A high degree of heterogeneity of actin isoforms was found in the COM and JUV stages, indicating the presence of many different isoforms of actins such as β-actin, actin cytoplasmic 3, and gamma-actin. In the PRECOM stage, the isoforms seemed to vary in heterogeneity and expression was barely visible. In the COM larvae isoforms, expression was significantly increased, whereas it was slightly decreased in JUV.

## 3.0. Discussion

In general, marine invertebrate larvae have evolved to undergo speedy metamorphosis to minimize the time that they are most vulnerable to predation, which is the period after losing their larval structures and before complete settlement in the new habitat [1]. In most spionid polychaetes, metamorphosis includes the loss of the larval swimming chaete, the redirection of the palps, and the development of a ciliated groove along the palps, all of which can be completed in a very short time [11]. In this study, we hypothesized that post-translational modification, particularly protein phosphorylation, is involved in regulating morphogenesis during larval metamorphosis. We observed a dramatic increase in the number of phosphorylated protein spots (282 spots), as shown in Figure 2B, and discovered 45 stage-specific total protein spots and 64 phosphoprotein spots (Figures 4C and 4D) during the competency period, which appears to support our hypothesis. The percentage of phosphorylation (76.4%) and the relative intensity of the phosphorylated spots was much higher in the COM stage than in the PRECOM (35.4%) and JUV (37.6%) stages. The detection of significantly more phosphoprotein spots in the COM larval stage may be due to the synthesis and storage of proteins while the larvae are attaining competency. Competent *P. vexillosa *larvae accumulate adult features, including neurochaetes, sensory structures (e.g., microvilli on the prostomium), and feeding structures (e.g., palps with ciliated grooves), when they undergo transient structural reorganization or when their organs begin to function. For example, their nuchal organs, which are originally located in the prostomium, gradually shift to the anterior segments on the dorsal side and are then replaced by adult organs after settlement and metamorphosis [12]. In fact, in over 40 species of spionidae, a pair of unpigmented photoreceptive organs reaches maximum size during the competent stage but then degenerates after metamorphosis [13]. We have reported a greater degree of phosphorylation in the COM stage of another polychaete, *Hydroides elegans *[14]. As competent polychaete larvae initiate habitat exploration and gain the ability to settle and metamorphose, we expect that specific phosphorylation or dephosphorylation serves a variety of functions required for metamorphosis, such as cue recognition, settlement signal transduction and amplification, and the preparation of juvenile tissues before attachment. The larger number of phosphoprotein spots up-regulated (32 spots) during competency and down-regulated (26 spots) during metamorphosis in *P. vexillosa *indicates that a dramatic change in the phosphoprotein expression profile occurs during larval metamorphosis (Figure 2C). When larvae metamorphose into juveniles, many proteins are probably degraded or they are no longer synthesized, as they are associated with "larval functions" that are no longer performed. Slightly higher degrees of protein phosphorylation have been found in the COM larval stage than in the JUV stage. For example, in the barnacle *Balanus amphitrite*, more phosphoprotein spots were detected in competent cyprids [15]. Likewise, in the bryozoan *Bugula neritina*, the highest percentage of phosphorylated protein spots and the highest overall phosphoprotein intensity were found in the competent swimming larvae that were ready to undergo metamorphosis [16]. Reversible phosphorylation is one of the essential switching mechanisms regulating enzyme activity, protein complex formation, protein degradation, and subcellular localization in higher organisms [17]. Chambon *et al. *[18] provided evidence of the transient phosphorylation of the JNK protein during the COM larval stage of the ascidian *Ciona intestinalis*, which renders JNK active and subsequently initiated attachment and metamorphosis. Phosphorylation signals in *C. intestinalis *are present only during the late swimming larval stage and they disappear before attachment.

In this study, cytoskeleton proteins and their isotypes (ACT, TUB, IFA, and MHC) were much more abundant in the COM larvae than in the PRECOM larvae (Tables 1 and 2), an observation confirmed by 2-D western blot analysis (Figure 9). These results suggest that cell and tissue assembly and the movement and disassembly of cytoskeleton proteins occur more frequently when larvae are attaining competency to settle, and that metamorphosis is mostly controlled by cytoskeletal dynamics [19,20]. The spatiotemporal expression of different tubulin isotypes may be related to a variety of physiological functions and post-translational modifications [21,22]. Subunits of tubulin are the building blocks of microtubules and are involved in many cellular processes, such as mitosis, cytokinesis, and cell division and migration [23-25]. Tubulin also forms the core structure of the cilia and thus contributes to the ciliation of all of the components of the "opposed-band feeding system" in polychaetes. A high abundance of tubulins has also been observed in pre-competent and competent larvae of the polychaete *Hydroides elegans *[14], but this abundance decreases dramatically in juveniles and adults, whereas in *P. vexillosa *tubulin and actin expression is lower in PRECOM larvae but increases during the competency period and metamorphosis, as shown by the 2-D Western blots (Figure 9). This can be explained by differences in behavior and morphological changes in the two species during metamorphosis. *P. vexillosa *experiences less structural loss during metamorphosis and does not require substantial development of juvenile organs [1], whereas in *H. elegans*, metamorphosis requires substantial tissue reorganization and dramatic morphological changes. IFAs are one of three types of cytoskeletal elements that work together with actin and microtubules to enhance structural integrity, cell shape, and cell and organelle motility [26]. The up-regulation of IFA in the COM larvae of *P. vexillosa *may provide resistance to deformation stress at different levels of development. In this study, several phosphorylated proteins were identified that may be related to the development of the larval muscle system. MHC is the motor protein of thick muscle filaments and its isoforms regulate muscle function [27].

MHC is also used as a marker of the physiological and developmental states of muscle [28], and it can be involved in the degeneration of the caudal muscle by apoptosis [29], the formation of new muscle (secondary myogenesis) [30], and the conversion of larval muscles to adult muscles [31]. The up-regulation of MHC in COM larvae and its subsequent down-regulation during metamorphosis may suggest the presence of a large proportion of larval myofibers that subsequently disappear during the processes of attaining competency and metamorphosis. Large amounts of myofibrillar proteins need to be synthesized to build up the muscles during metamorphosis.

PM is the main structural component of the thick filament of smooth muscles. A high abundance of PM in COM larvae of *P. vexillosa *may indicate that larval metamorphosis is accompanied by a massive reorganization of striated muscles, followed by the development of smooth muscles. The abundant expression of TP, actin-binding protein, could be due to muscle degeneration and differentiation at the onset of larval metamorphosis [32,33].

Of the four phosphoproteins (EP1, GRX 3, GA2-PA, and AFABP) that are up-regulated in the COM stage, three are involved in metabolism, immune defense, and energy regulation. EP1 is a bifunctional enzyme from the hydrolase superfamily. It is mainly involved in the amino-acid biosynthesis pathway [34]. The up-regulation of this phosphoprotein in COM larvae of *P. vexillosa *may be related to the phosphatase/enolase activity of this protein during metamorphosis. Grx belongs to a family of low molecular weight thiol-disulfide oxidireductases involved in cellular functions such as DNA synthesis, the generation of reduced sulfur and signal transduction [35]. GRX 3 acts as an antioxidant and provides defense against oxidative stress, which enhances the organism's ability to withstand subsequent stress [36]. The up-regulation of GRX 3 in COM larvae may be related to exposure to environmental stressors, pathogens, and stress generated from constant habitat-searching behavior. To counterbalance this stress, a series of protective responses are triggered in the larvae. In our previous studies, we found that protective responses against oxidative stress were also triggered in *B. neritina *larvae, as shown by the up-regulation of HSP expression during larval settlement [37]. Interestingly, GA-2 PA is the RNA binding domain involved in post-transcriptional gene expression processes, including those of mRNA and rRNA [38], and it is up-regulated in the COM stage in *P. vexillosa*. However, the biological function and physiological significance of this protein remain unclear. Fatty acid binding proteins (FABP) are ubiquitous proteins that are believed to be involved in intracellular fatty acid transport and metabolism [39]. AFABP is a cytosolic fatty acid chaperone expressed in adipocytes, and it serves as a major fat storage site and regulator of energy balance and inflammation [40]. The up-regulation of AFABP in COM larvae may be related to the requirement of a large amount of energy storage for settlement and metamorphosis.

## Conclusion

This is the first detailed study to combine affinity enrichment of phosphoproteins, 2D-PAGE, and mass spectrometry to investigate phosphoprotein expression during larval metamorphosis in a polychaete. The results show differential expression or changes in the phosphorylation level of proteins in the larval-juvenile transition. In particular, a higher percentage of phosphorylation, a greater number of specific phosphoproteins, and a greater abundance of phosphorylated proteins in the competent larvae stage are observed. Notably, most of the phosphoproteins in high abundance were different isoforms of cytoskeleton proteins, which suggests the probable role of microtubule dynamics in larval metamorphosis. Overall, most of the identified phosphoproteins are involved in cell differentiation, development, transcription, metabolism, and protein-protein interaction, suggesting their possible active roles in the regulatory mechanisms of larval metamorphosis in spionid polychaetes.

## 4.0. Methods

### 4.1. Larval culture and sample collection

Adult individuals of the spionid *P. vexillosa *were collected from subtidal soft-bottom substrates in Sai Kung, Hong Kong (22°25'N, 114°17'E) and maintained in laboratory cultures. From the adult cultures, pre-competent larvae (PRECOM), competent larvae (COM), and newly metamorphosed juveniles (JUV) (Figure 1) were collected according to the procedure laid out by Mok *et al*. [11]. PRECOM larvae with 11-13 setigers were collected on day 5 and COM larvae with 12-14 setigers were collected on day 6 or 7. Some of the COM larvae were transferred to autoclaved sediment with a particle size of less than 60 μm for 2 hr until they settled and underwent metamorphosis. The resulting JUV were collected by wet sieving and probing of the sediment tubes. After collection, the samples of each developmental stage were briefly rinsed with autoclaved filtered seawater (AFSW) and fixed in a lysis buffer consisting of 7 M urea, 2 M thiourea, 4% 3-[(3-Cholamidopropyl) dimethylammonio]-2-hydroxy-1-propanensulfonate (CHAPS), 1% 1, 4 dithiothreitol (DTT), and protease and phosphatase inhibitors (Roche, Basel, Switzerland). The samples were frozen at -80°C for less than one week before being used for protein extraction. Three independently prepared replicates of each developmental stage were used for the proteomic analysis.

### 4.2. *P.vexillosa *transcriptome database construction

Two RNA pools were prepared for the *P. vexillosa *transcriptome profiling. For the first RNA pool, total RNA was extracted from the competent larval stage by using Trizol Reagent (Invitrogen, Carlsbad, CA, USA) and mRNA was amplified by using MessageAmp™ II aRNA amplification kit (Ambion, Austin, TX, USA). For second mRNA pool, total RNA was extracted from a mixed sample pool of newly hatched larvae, pre-competent larvae, competent larvae, and the adult and mRNA was extracted by using Poly(A) purist™ kit (Ambion). The cDNA systhesis was performed from both mRNA pools by using SuperScript double strand cDNA synthesis kit (Invitrogen, Carlsbad, CA, USA) with random priming. The 454 pyrosequencing were performed according to standard protocol. The sequence assembly were performed by using Newbler software 2.3 version (Roche, Nutley, NJ, USA). The gene prediction and annotation were performed according the method described by Wang et al [41].

### 4.3. Preparation of protein samples and 2-DE

Sample preparation was carried out according to the procedure described by Mok *et al*. [6], with slight modifications. First, the samples were sonicated (Branson Digital Sonicator 250) on ice using ten 5 s blasts of 15% amplitude with 10 s pauses between blasts. The samples were then centrifuged at 13,000 rpm for 20 min, and the supernatant was desalted and purified using a 2-DE cleanup kit (BioRad, Hercules, CA, USA) to remove any polysaccharide contaminants. The purified protein pellets were resolubilized in lysis buffer (7 M of urea, 2 M of thiourea, 4% CHAPS, 1% DTT), and the protein concentration was determined using the modified Bradford method [42]. Before rehydration, 750 μg of each protein sample was sonicated for 10 min, vortexed, incubated at room temperature for 2 hr, and then sonicated again for 10 min to enhance the protein solubilization. Rehydration was carried out using 300 μl of sample in rehydration buffer (7 M of urea, 2 M of thiourea, 4% CHAPS, 40 mM of DTT, 0.5% pI 4-7 ampholyte, and 1% bromophenol blue) on 17 cm immobilized pH gradient (IPG) strips (pH 4-7) for ~ 14 h. The samples were then subjected to isoelectrical focusing (IEF) using a Protean IEF Cell (BioRad, Hercules, CA, USA). Focusing was carried out at 250 V for 20 min, and then along a gradient from 1,000 to 8,500 V over 2 hr to give a total 60,000 Vh. The current did not exceed 50 mA per strip. After IEF, reduction and alkylation of the IPG strips were carried out using DTT and iadoacetamide (IAA), and two-dimensional SDS-PAGE was performed following the protocol described by Qian *et al. *[43].

### 4.4. Gel imaging and analysis

The gels were fixed overnight in a fixing solution of 40% methanol and 10% acetic acid, and sequentially stained with Pro-Q Diamond phosphoprotein gel stain (Invitrogen, CA, USA) and Sypro Ruby total protein gel stain (Invitrogen, CA, USA). They were then incubated for 2 hr in ProQ Diamond stain, followed by destaining with 20% ACN in 50 mM of sodium acetate (pH 4.0) for 3 hr. After destaining, the gels were rinsed with deionized water and scanned for phosphoprotein spots using a Typhoon trio imager (GE Healthcare, Piscataway, NJ, USA) at an excitation of 532 nm with a 610 BP 30 emission filter. After the scan images were acquired, the gels were incubated overnight in the dark with Sypro Ruby protein stain and scanned again using the Typhoon trio imager at an excitation of 582 nm with a 610 BP 30 emission filter.

The three replicate gels stained with phosphoprotein stain and total protein stain were grouped accordingly and compared. The spot intensities were normalized such that the total density of each image was equal. Quantitative and qualitative analyses were carried out using the PDQuest software (BioRad, Hercules, CA, USA). Only spots that were present in all three replicate gels were considered. A two-fold threshold was set for the quantitative detection of protein changes between stages. Phosphoprotein spots that were significantly different (Student's *t*-test, *p *< 0.01) in successive stages were considered to be up- or down-regulated.

### 4.5. Phosphoprotein enrichment and 2-DE

Affinity capture of phosphoproteins from the PRECOM larvae, COM larvae, and JUV was performed using a Pro-Q Diamond Phosphoprotein Enrichment Kit (Invitrogen, Oregon, USA) according to the manufacturer's protocol with minor modifications as described by Makrantoni *et al. *[10]. In brief, 1 mg of protein extract was prepared using lysis buffer supplemented with a phosphotase inhibitor and purified with a 2DE cleanup kit. The precipitated protein pellet was resolubilized in lysis buffer. The protein solutions were diluted with 5 ml of washing buffer and applied to a column containing 1 ml of resin. After washing the column with a wash buffer, bound proteins were eluted with 250 μl of elution buffer. The elution step was repeated five times. The elution was concentrated until the sample volume was reduced to approximately 50 μl using Vivaspin filtration concentrators. A solution of 25 mM of Tris, pH 7.5, and 0.25% CHAPS was then added to the retention reservoir, and the sample was concentrated to a volume of approximately 50 μl. The samples were precipitated using the methanol-chloroform-water method and the precipitates dissolved in 300 μl of rehydration buffer. After rehydration, the enriched phosphoproteins were subjected to IEF and 2-DE following the protocol described by Qian *et al. *[43].

### 4.6. Mass spectrometry

Selected abundant phosphoprotein spots and differentially expressed spots (See Additional file 1) on phosphoprotein-enriched 2-DE gels were excised, washed, and digested in 20 μL of 12.5 ng/mL trypsin (Promega, Madison, WI, USA) in 10% acetonitrile and 10 mM of NH_4_HCO_3 _at 37°C for 16 hr. The peptides were extracted following the protocol described by Qian *et al. *[43] and dried in a speed vacuum. The peptides were dissolved in 3 μl of 0.1% trichloroacetic acid and 3 μl of each sample was spotted on an AnchorChip PAC 384 HCCA (Bruker Daltonics, Bellirica, MA, USA) target plate pre-coated with a matrix of cyano-4-hydroxy-cinnamic acid, followed by desalting with 10 mM of ammonium phosphate in 0/1% TFA. The samples were analyzed using an Ultraflex III TOF/TOF mass spectrometer (Bruker Daltonics, Bellirica, MA, USA) as described in [4]. External calibration was performed using Bruker peptide calibration standards. Mass spectra (MH+) were acquired by FlexControl (version 3.0, Bruker Daltonics), which recorded in the range 800-3500 Da. The MS/MS information was obtained in LIFT (laser-induced forward transfer) mode. An in-house database was constructed with transcriptome sequences of *P. vexillosa*. The MS and MS/MS spectra were combined using the BioTools software (version 3.1, Bruker Daltonics) and searched against the in-house database using the MASCOT software (Matrix Science). The search parameters were set as 50 ppm for the PMF peptide tolerance and 0.2 Da for the MS/MS tolerance. The combined spectra were also searched against the NCBInr database to obtain more information. Search results from the combined spectra that were statistically significant (*p *< 0.05) were accepted.

### 4.7. 2-DE Western Blot

2-DE western blot analysis was performed to confirm the abundance of the tubulin and actin isoforms following the protocol described in [14]. Equal amounts of lysates (100 μg) from each developmental stage were subjected to isoelectric focusing using 7 cm IPG strips with a linear pH 4-7 gradient (Bio-Rad, Hercules, CA, USA), and then electrophoretically separated on 12.5% (8 × 7.3 cm2) SDS-PAGE and transferred onto a Hybond ECL nitrocellulose membrane (Amersham, Buckinghamshire, UK). After blocking, the membranes were incubated for 3 hr at room temperature with antibodies of anti-tubulin (Cell Signaling, Danvers, MA, USA) and anti-actin (Millipore, Billerica, MA USA) at a dilution of 1:1000. The membranes were then incubated with corresponding HRP conjugated secondary antibodies at a dilution of 1:5000 for 1 hr and then developed using an ECL western blotting analysis system (Millipore, Billerica, MA USA).

## Authors' contributions

PYQ conceptualized the study and revised the manuscript; KHC performed the sample preparation, 2DE, protein enrichment, MALDI-TOF MS analysis, Western blot analysis and drafted the initial manuscript; FSM carried out the larval culture and collection and participated in the sample preparation, protein enrichment, data analysis, and preparation of the figures; WH performed 454 pyrosequencing and transcriptome database construction.

All of the authors read and approved the final version of the manuscript.

## Supplementary Material

Additional file 1 **Enriched phosphoprotein profile of competent larvae of *P. vexillosa***. The phosphoproteins were enriched and separated on 7 cm IPG strips (pH 4-7) followed by 2-DE. The gel was stained with the Pro-Q Diamond phosphoprotein gel stain, and then post-stained for total protein with the SYPRO Ruby protein gel stain and colloidal Coomassie blue. Abundant phosphoproteins (marked with an arrow) and differentially expressed proteins (marked with a circle) were identified by mass spectrometry. Click here for file
