# Supplementary figures and images for: Phosphoproteome analysis during larval development and metamorphosis in the spionid polychaete Pseudopolydora vexillosa
Source: BMC Dev Biol. 2011 May 25;11:31. doi: 10.1186/1471-213X-11-31 (PMC3115903; doi:10.1186/1471-213X-11-31)

## Slide 1
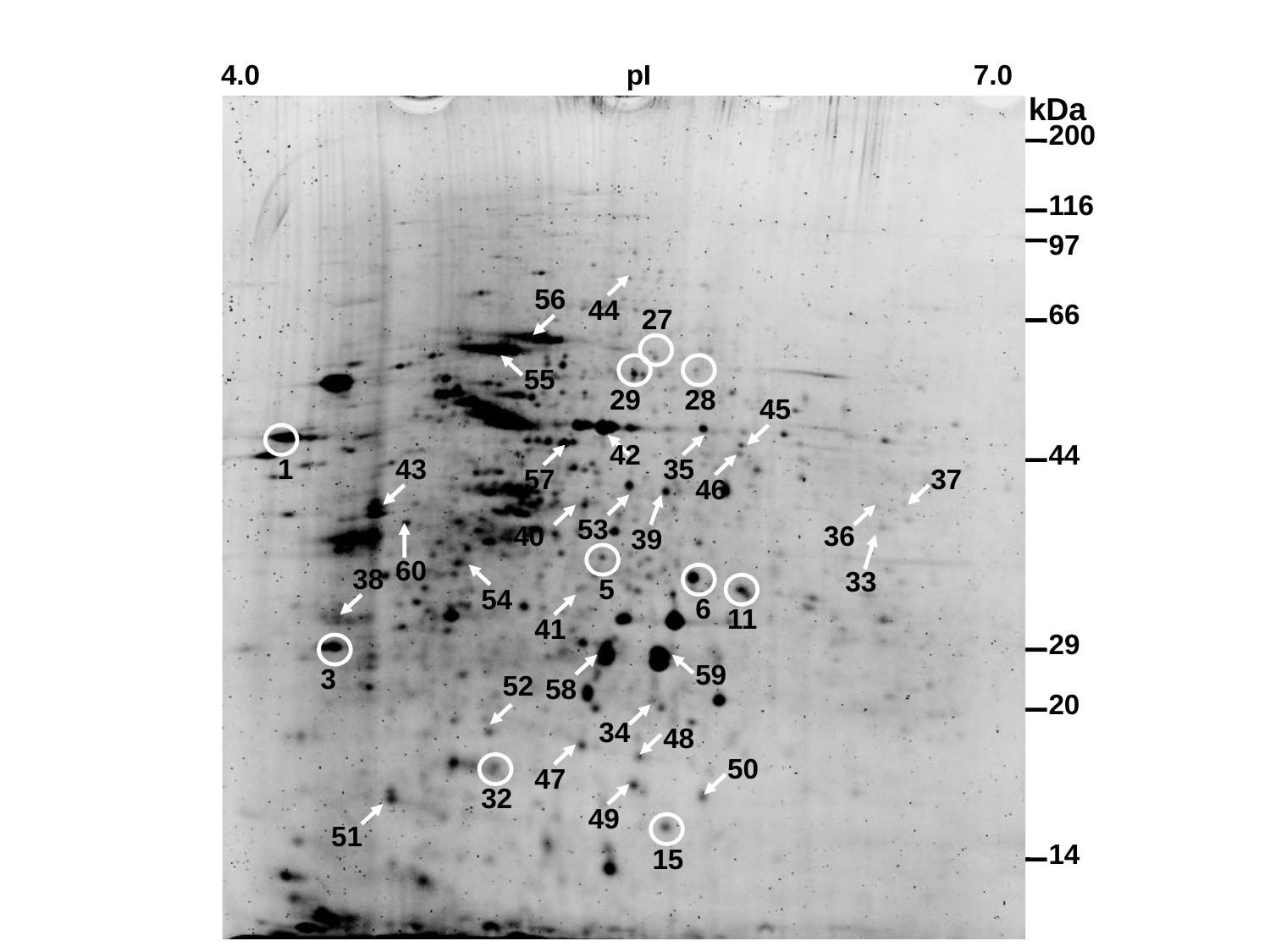

4.0
pI
7.0
56
44
27
55
29
28
45
42
1
43
35
57
37
46
53
40
36
39
60
38
33
5
54
6
11
41
59
3
52
58
34
48
50
47
32
49
51
15
kDa
200
116
97
66
44
29
20
14

Supplement: Additional file 1 — Enriched phosphoprotein profile of competent larvae of P. vexillosa. The phosphoproteins were enriched and separated on 7 cm IPG strips (pH 4-7) followed by 2-DE. The gel was stained with the Pro-Q Diamond phosphoprotein gel stain, and then post-stained for total protein with the SYPRO Ruby protein gel stain and colloidal Coomassie blue. Abundant phosphoproteins (marked with an arrow) and differentially expressed proteins (marked with a circle) were identified by mass spectrometry. [file 1471-213X-11-31-S1.PPT]
